# Supplementary material for: From sequence to enzyme mechanism using multi-label machine learning
Source: BMC Bioinformatics. 2014 May 19;15:150. doi: 10.1186/1471-2105-15-150 (PMC4229970; doi:10.1186/1471-2105-15-150)
Supplement: Additional file 2 — Java code of ml2db. Additional file ml2db_code.tar.gz contains the Java source code to run the multi-label machine learning experiments and save the results to database. The code’s Javadoc is included. [file 1471-2105-15-150-S2.zip › additional file 2/ml2db/utils/doc/uk/ac/ed/inf/utils/maputils/package-use.html]

Uses of Package uk.ac.ed.inf.utils.maputils


---


|  |  |  |
| --- | --- | --- |
| **Overview** \| **Package** \| Class \| **Use** \| **Tree** \| **Deprecated** \| **Index** \| **Help** | |  |
| PREV   NEXT | **FRAMES**    **NO FRAMES**     **All Classes** |  |


---


## **Uses of Package uk.ac.ed.inf.utils.maputils**

| Packages that use uk.ac.ed.inf.utils.maputils | |
| --- | --- |
| **test.maputils** |  |
| **uk.ac.ed.inf.utils.collectionutils.setutils** |  |
| **uk.ac.ed.inf.utils.database** |  |
| **uk.ac.ed.inf.utils.maputils** |  |

Classes in uk.ac.ed.inf.utils.maputils used by test.maputils

****IndexedOneToManyMap****
  
          A one-to-many map that uses an internal integer index to save memory.

****OneToManyMap****
  
          A map containing for each key a set of values.

****TableMap****
  
          A table map is a map containing relations [key > list of values] [value >
list of keys]

Classes in uk.ac.ed.inf.utils.maputils used by uk.ac.ed.inf.utils.collectionutils.setutils

****OneToManyMap****
  
          A map containing for each key a set of values.

Classes in uk.ac.ed.inf.utils.maputils used by uk.ac.ed.inf.utils.database

****OneToManyMap****
  
          A map containing for each key a set of values.

Classes in uk.ac.ed.inf.utils.maputils used by uk.ac.ed.inf.utils.maputils

****OneToManyMap****
  
          A map containing for each key a set of values.

---


|  |  |  |
| --- | --- | --- |
| **Overview** \| **Package** \| Class \| **Use** \| **Tree** \| **Deprecated** \| **Index** \| **Help** | |  |
| PREV   NEXT | **FRAMES**    **NO FRAMES**     **All Classes** |  |


---
